# Seroprevalences of autoantibodies and anti-infectious antibodies among Ghana’s healthy population

**DOI:** 10.1038/s41598-020-59693-x

**Published:** 2020-02-18

**Authors:** Itai Katz, F. De Luca, Bartholomew Dzudzor, Baffour Kyei Sarpong, Beatrice Osei-Appiah, Danielle Azoulay, Daphna Katz, Dzifa Dey, Boris Gilburd, Howard Amital, Sandro Vento, Yehuda Shoenfeld, Ora Shovman

**Affiliations:** 1 Zabludowicz Center for Autoimmune Diseases, Sheba Medical Center, Tel Hashomer, Israel; 2 Sackler Faculty of Medicine, Tel Aviv University, Tel Aviv, Israel; 3 Department of Allergology and Immunology, Niguarda Ca’ Granda Metropolitan Hospital, Milan, Italy; 4 Department of Medical Biochemistry, University of Ghana School of Medicine and Dentistry, College of Health Sciences, Korle-Bu, Accra Ghana; 5 School of Medical Sciences, University of Sydney, New South Wales, Australia; 6 Assuta Ashdod Medical Center, Ashdod, Israel; 7 Department of Medicine and Therapeutics, University of Ghana School of Medicine and Dentistry, College of Health Sciences, Korle-Bu, Accra Ghana; 8 Department of internal medicine ‘B’, Sheba Medical Center, Tel Hashomer, Israel; 9 Faculty of Medicine, University of Puthisastra, Phnom Penh, Cambodia; 10 I.M. Sechenov First Moscow State Medical University of the Ministry of Health of the Russian Federation (Sechenov University), Moscow, Russia; 11 Past Incumbent of the Laura Schwarz-Kipp Chair for Research of Autoimmune Diseases, Sackler Faculty of Medicine, Tel Aviv University, Tel Aviv, Israel

**Keywords:** Viral infection, Epidemiology

## Abstract

Autoantibodies, which are antibodies that target self-epitopes, have considerable diagnostic, prognostic and predictive value in specific autoimmune diseases. Various infectious agents have been linked *via* numerous mechanisms to the formation of different autoantibodies. Therefore, estimating the prevalence of autoantibodies and anti-infectious antibodies in different populations is of high importance. Different genetic and environmental pressures, such as these found in Ghana’s different geographical provinces, may affect the prevalence of autoantibodies. In this study, we assessed the seroprevalence of a diverse panel of autoantibodies and anti-infectious antibodies among the healthy Ghanaian population and investigated possible environmental and genetic predispositions for autoantibodies and autoimmunity. The sera of 406 healthy individuals were obtained from Greater Accra, Upper West, Eastern and Volta regions. Multiplexed assay and chemiluminescent immunoassay techniques were utilized to assess the presence of a panel of autoantibodies and anti-infectious antibodies. We found a high prevalence of anti-HSV-1 IgG (91–100%), anti-EBNA IgG (81–93%) and anti-EBV-VCA IgG (97–100%) antibodies. The prevalence of ANA (at least one of: anti-dsDNA; anti-chromatin; anti-ribosomal-P; anti-Ro/SSA; anti-La/SSB; anti-centromere B; anti-Sm; anti-Sm/RNP; anti-Scl-70; anti-Jo1; anti-DFS70) was estimated at 14%. An inverse association between anti-HSV-2 antibodies and ANA (p = 0.044; adjusted OR = 0.398; CI [0.162–0.975]) was found, after adjusting for differences in gender, age, and familial history of autoimmune diseases. A trend towards reduced seroprevalence of anti-dsDNA antibodies among subjects who were positive for anti-HSV-2 antibodies was also noted (p = 0.1). In conclusion, the inverse association between anti-HSV-2 antibodies and ANA positivity suggests a possible protective role of HSV-2 infection against autoimmunity.

## Introduction

Currently, the etiology of autoimmune diseases (AIDs) is not completely understood. Many variables are thought to play a role in the development of these diseases, including genetic, immunological, hormonal and environmental factors^1^. These various factors and the interactions between them, which constitute the “Mosaic of Autoimmunity”, may contribute to autoimmunity by different mechanisms. One of these mechanisms is associated with the loss of self-tolerance^2^.

Infectious agents, which are members of the environmental pebble of the mosaic, can induce loss of tolerance through various mechanisms, such as: (1) Epitope spreading, i.e. development of an autoimmune response mediated by autoreactive T-cells and expansion of the auto-reactive B-cell repertoire to endogenous epitopes, following the release of self-antigens during an inflammatory response^3^; (2) Molecular mimicry, that occurs when foreign antigens share sequence or structural similarities with self-antigens; the immune responses can be directed against peptides with similar charge distribution and overall shape^4,5^. Segal *et al*.^6^, in particular, have recently described an association between human papillomavirus (HPV) infection and systemic lupus erythematosus (SLE); (3) Bystander activation, where a microbial infection stimulates toll-like receptors (TLRs) and other pattern recognition receptors on antigen-presenting cells (APCs), resulting in the production of pro-inflammatory mediators, which in turn may lead to tissue damage^7^; (4) Chronic infections, such as Epstein-Barr virus (EBV) or hepatitis C virus (HCV) infections, induce constant activation and proliferation of T-cells as well as B-cells, which in turn can produce polyclonal and monoclonal antibodies and immune complexes, which might lead to loss of self-tolerance^8^.

In the healthy population, a diverse repertoire of circulating antibodies may be detected. A proportion of these antibodies react with self-antigens; thus, they are referred to as autoantibodies^1^. These autoantibodies have considerable diagnostic, prognostic and predictive value in specific AIDs, and are also used for the classification of different AIDs.

In the last decades, several studies have shown that the presence of certain autoantibodies precedes the onset of some AIDs^7–10^. For instance, rheumatoid factor (RF) and/or anti-cyclic-citrullinated-peptide antibodies (anti-CCP) were detected in serum samples obtained a median of 4.5 years before the onset of rheumatoid arthritis (RA), with a sensitivity of 49% and a specificity as high as 98–99%^9^. In SLE, the presence of antibodies against ribonucleoproteins (RNP), Smith antigen (Sm), double stranded DNA (dsDNA), cardiolipin, Ro/SSA, and La/SSB preceded clinical diagnosis by 7–10 years in 88% of the subjects^10^.

The possible prediction of AIDs is complicated by the vast differences in the circulating autoantibody repertoire between ethno-geographic clusters. Shapira *et al*.^11^ described and deciphered differences in the prevalence of both autoantibodies and anti-infectious antibodies, among six healthy groups of various geographic regions. Notably, anti-nuclear antibodies (ANA) were positive in 45% of Colombians compared with a 12% prevalence among Italians and Dutch, and an 11% positivity in Israelis. The authors also observed differences in the prevalence of antibodies against different infectious agents, with higher positivity rates of antibodies against *T. pallidum* and *H. pylori* among subjects of Papua New Guinea and Colombians respectively. Thus, the high exposure to infections might partially explain the higher prevalence of autoantibodies in specific geographic regions^11–13^. This study aims to analyze the variance in the prevalence of autoantibodies and anti-infectious antibodies among healthy subjects from different regions of Ghana, a West African country. To the best of our knowledge, this is the first study that simultaneously evaluated the presence of specific autoantibodies and anti-infectious antibodies in the healthy Ghanaian population.

## Materials and Methods

### Study design

This study is a cross-sectional study which describes and compares the seroprevalence of autoantibodies and anti-infectious antibodies among healthy subjects from different geographical regions of Ghana.

### Study population

**Figure 1:**
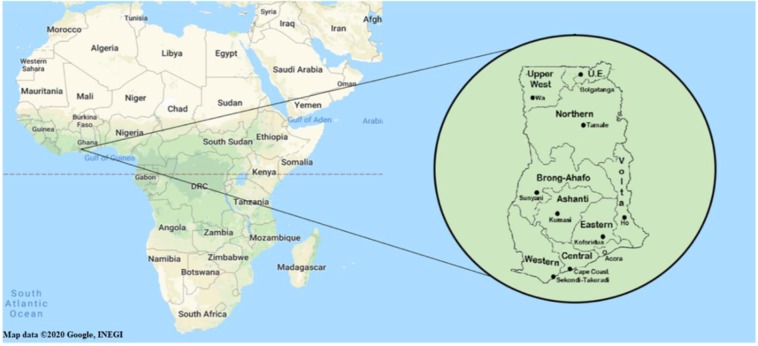
A map of Africa detailing the division of Ghana to the different regions. Region 1 - Greater Accra region. Region 2 - Upper West region. Region 3 - Eastern region. Region 4 - Volta region.

The sera of 406 randomly selected healthy volunteers of at least 18 years of age were collected from four different geographical regions in Ghana (see Fig. 1 for a detailed map). Specifically: 81 subjects from Greater Accra region (R1), 71 subjects from Upper West Ghana (R2), 81 subjects from the Eastern region (R3), and 173 subjects from the Volta region (R4).

### Ethics

This study was conducted in accordance with the provisions of the Declaration of Helsinki and Good Clinical Practice guidelines. The Institutional Review Board of the College of Health Sciences, University of Ghana approved the protocol. Data were collected at each site and monitored. All patients received an oral and written explanation and gave informed consent to be included in this research.

### Laboratory procedures

#### Multiplexed assay

Using a fully automated multiplexed platform - Bioplex 2200 system (Bio-Rad Bioplex 2200 system, Bio-Rad Laboratories Hercules, CA USA) - we measured the following antibodies:

1. ANA, including: anti-dsDNA; anti-chromatin; anti-ribosomal-P; anti-Ro/SSA 52; anti-Ro/SSA60; anti-La/SSB; anti-centromere B; anti-Sm; anti-Sm/RNP; anti-Scl-70; anti-Jo1.
2. Anti-phospholipid antibodies, including: anti-cardiolipin (aCL) (IgM, IgG, IgA); and anti-β2-Glycoprotein 1 (anti-b2GPI) (IgM, IgG, IgA).
3. Anti-infectious antibodies: EBV viral capsid antigen (EBV-VCA) IgG and IgM; EBV nuclear antigen (EBNA) IgG, EBV early antigen (EBV-EA) IgG; EBV Heterophile (EBV-H) IgM; Herpes Simplex Virus 1 (HSV-1) and 2 (HSV-2) IgG.

The Bio-Rad Bioplex 2200 system utilizes multiplex flow immunoassay to detect and identify multiple antibodies to different antigens in a single incubation. The sera of the subjects (5 µl) were diluted and incubated with a reagent containing distinctly colored bead sets, created by the use of two fluorescent dyes at distinct *ratios* and coated with different antigens. After incubation and a wash cycle, an anti-human IgG or IgM antibodies conjugate with phycoerythrin was added. In the following step, the beads were passed through a detector that identified the fluorescence intensity. The quantity of antibodies “captured from antigen” was determined by the fluorescence of an anti-human IgG or IgM-phycoerythrin-labeled conjugate. Furthermore, the relative fluorescence intensity (RFI) was normalized to an antibody index. Additional details are described elsewhere^14^.

The cut-off values for the autoantibodies included in the ANA test coincided with the manufacturer’s recommended values (1.0 antibody index (AI)), except for anti-dsDNA antibodies (5 AI). The cut-off values for the anti-phospholipid antibodies were: 15 GPL/ml for anti-b2GPI IgG, 15 MPL/ml for anti-b2GPI IgM, and 15 Units/ml for anti-b2GPI IgA and for aCL (IgM, IgG, IgA). The cut-off value for the anti-infectious antibodies was 1.1 AI.

#### Chemiluminescent immunoassay

The anti-dense fine speckled 70 (anti-DFS-70) antibodies were tested using a Chemiluminescent immunoassay (CLIA, Bio Flash INOVA, San Diego CA). This method utilized paramagnetic beads coated with the different antigens. After incubation with the sera and washings, the bound antibodies were identified by an anti‐IgG-IgM antibody linked to an isoluminol derivative. Afterwards, the starter reagents were added, and a flash chemiluminescence reaction was generated. The light signal was measured in a photomultiplier, indicating the presence of IgM or IgG antibodies. In our study, we incubated the samples of the patients with the paramagnetic beads, which were coated with the antigens listed above. Following that, the beads were washed to remove any unbound antibodies. Immediately after the wash, a tracer was added for a second incubation. After the second incubation, the beads were washed again. Finally, the chemiluminescent reaction took place, by adding peroxide solutions. The light produced from the reaction was measured as Relative Light Units (RLUs), by an optical system. The RLUs are proportional to the concentration of isoluminol conjugate bound to the human IgG or IgM antibodies. Additional details are described elsewhere^15^.

ANA positivity was defined as a positive result for any of the autoantibodies subsumed as “ANA” by the multiplexed assay and/or anti-DFS70 positivity by CLIA.

### Statistical analysis

The prevalence of autoantibodies and anti-infectious antibodies, and their corresponding Fisher’s 95% confidence intervals (CI), was estimated using Winpepi Version 11.65 (Abramson, J.H. WINPEPI updated: computer programs for epidemiologists, and their teaching potential. Epidemiologic Perspectives & Innovations 2011, 8:1).

The prevalence of the autoantibodies and the anti-infectious antibodies among the different Ghana regions was compared initially by utilizing the Kruskal-Wallis test and after that by either Chi-square test or Fisher’s exact test. Autoantibodies and anti-infectious antibodies of interest were tested for association using either Chi-square test or Fisher’s exact test. Similarly, Chi-square test or Fisher’s exact test and Kruskal-Wallis test were used to assess different variables and their association to ANA. Lastly, a binary multivariable model was built with an insert approach to encompass various confounders. Considering the variance in demographics and clinical status of our study population, several possible relevant confounders were included in our multivariate analysis regardless of significance that was determined by the aforementioned methods. Thus, we controlled for gender, age, region, and familial history of autoimmune diseases. Additionally, SPSS (IBM SPSS Statistics for Macintosh, Version 25.0. Armonk, NY: IBM Corp) was used for Chi-square test, Fisher’s exact test, Kruskal-Wallis test as well as rendering plots. P values less than 0.05 were considered significant.

## Results

The study included 406 healthy subjects from 4 regions of Ghana. The demographic and clinical characteristics vary between the groups and are detailed in Table 1.

**Table 1:** Demographic and clinical characteristics of the study population, N (%).

Variable	Greater Accra	Upper West	Eastern	Volta	Total
	(N = 81)	(N = 71)	(N = 81)	(N = 173)	(N = 406)
**Age group (years)**					
18–30	11 (13.6)	50 (70.4)	9 (11.1)	60 (34.7)	130 (32)
31–40	16 (19.8)	9 (12.7)	14 (17.3)	25 (14.5)	64 (15.8)
41–50	21 (25.9)	8 (11.3)	13 (16)	16 (9.2)	58 (14.3)
51–60	10 (12.3)	2 (2.8)	24 (29.6)	29 (16.8)	65 (16)
*≥*60	12 (14.8)	1 (1.4)	21 (25.9)	19 (11)	53 (13.1)
Missing	11 (13.6)	1 (1.4)	0 (0)	24 (13.9)	36 (8.9)
**Gender**					
Female	22 (27.2)	38 (53.5)	14 (17.3)	59 (34.1)	133 (32.8)
Male	50 (61.7)	33 (46.5)	67 (82.7)	91 (52.6)	241 (59.4)
Missing	9 (11.1)	0 (0)	0 (0)	23 (13.3)	32 (7.9)
**Ethnicity**					
Akan	8 (9.9)	3 (4.2)	55 (67.9)	25 (14.5)	91 (22.4)
Ga-Adangbe	48 (59.3)	0 (0)	21 (25.9)	3 (1.7)	72 (17.7)
Ewe	3 (3.7)	0 (0)	5 (6.2)	103 (59.5)	111 (27.3)
Northern	7 (8.6)	68 (95.8)	0 (0)	19 (11)	94 (23.2)
Missing	15 (18.5)	0 (0)	0 (0)	23 (13.3)	38 (9.4)
**AD-FHx**					
No	55 (67.9)	66 (93)	59 (72.8)	135 (78)	315 (77.6)
Yes	17 (21)	5 (7)	22 (27.2)	15 (8.7)	59 (14.5)
Missing	9 (11.1)	0 (0)	0 (0)	23 (13.3)	32 (7.9)

AD, Autoimmune disease; FHx, Family medical history.

With regard to anti-infectious antibodies’ seroprevalence in the different regions (Table 2), we found a high seroprevalence of anti-EBNA IgG antibodies (81–93%) and anti-EBV-VCA IgG (97–100%). Similarly, anti-HSV-1 IgG antibodies were highly prevalent (91–99%). The seroprevalence of anti-HSV-2 IgG was variable and ranged from 16% to 63% in the different regions. In contrast, a relatively low seroprevalence of anti-EBV-EA IgG antibodies (6–11%) and anti-EBV-VCA IgM antibodies (5–20%) were found. Interestingly, anti-EBV-H antibodies were not prevalent.

**Table 2:** Seroprevalences of anti-infectious antibodies. % (95% CI).

Variable	Greater Accra	Upper West	Eastern	Volta	Total
**Herpes simplex**					
HSV1 IgG	98.8 (93.2–100)	91.4 (82.3–96.8)	97.4 (91–99.7)	97.8 (93.6–99.5)	96.7 (94.3–98.3)
HSV2 IgG	40 (29.2–51.6)	15.7 (8.1–26.4)	62.8 (51.1–73.5)	21.5 (14.9–29.4)	33.3 (28.5–38.4)
**Epstein-Bar virus**					
EBV-EA IgG	11.3 (5.3–20.3)	7 (2.3–15.7)	6.4 (2.1–14.3)	6.3 (3.1–11.3)	7.5 (5.1–10.6)
EBV-VCA IgM	5 (1.4–12.3)	20 (11.4–31.3)	12.1 (4.5–19.2)	20 (13.6–27.8)	13 (9.7–16.8)
EBV-VCA IgG	100 (95.5–100)	98.6 (92.4–99.7)	97.4 (91–99.7)	100 (97.7–100)	99.2 (97.8–99.8)
EBNA IgG	90 (81.2–95.6)	93 (84.3–97.7)	80.8 (70.3–88.8)	93.1 (88–96.5)	90 (86.5–92.6)
EBV-H IgM	0 (0–4.5)	0 (0–5.1)	0 (0–4.6)	0 (0–4)	0.3 (0–1.5)

CI, Confidence interval; HSV1/2, Herpes simplex Virus type 1/2; IgG/M, Immunoglobulin G/M; EBV, Epstein-Bar virus; EA, Early antigen; CA, Capsule antigen; NA, Nuclear antigen (EBNA); H, Heterophile.

With regard to the autoantibodies’ seroprevalence in the different regions (Table 3), we found that 14% of healthy Ghanaian population were ANA positive. The results for specific autoantibodies were as follows: anti-dsDNA, 0–6%; anti-chromatin, 0–1.5%; anti-Ro, 0–1.2%; anti-La 0–4.7%; anti-centromere-B, 0–2.6%; anti-Sm, 0–1.5%; anti-sm/RNP, 3.7–10.5%; anti-Scl-70, 0–1.8%; anti-Jo-1, 0–1.3%; anti-DFS-70, 0–1.3%.

**Table 3:** Seroprevalences of autoantibodies. % (95% CI).

Variable	Greater Accra	Upper West	Eastern	Volta	Total
**Anti-nuclear antibodies**					
ANA	6.9 (2.3–15.5)	16.4 (8.5–27.5)	11.5 (5.4–20.8)	17.8 (12.3–24.4)	14.3 (10.9–18.1)
**Specific autoantibodies**					
dsDNA	0 (0–4.8)	6 (1.7–14.6)	0 (0–4.6)	3 (0.1–6.9)	2.3 (1.1–4.3)
Chromatin	0 (0–4.8)	1.5 (0–8)	0 (0–4.6)	0 (0–2.2)	0.3 (0–1.4)
Ribosomal-P	0 (0–4.9)	0 (0–5.4)	0 (0–4.6)	0 (0–2.2)	0 (0–1)
Ro/SSA(52 and/or 60)	0 (0–4.5)	0 (0–5.4)	0 (0–4.6)	1.2 (0.1–4.2)	0.5 (0.1–1.8)
La/SSB	1.3 (0–7.2)	0 (0–5.4)	2.6 (0.3–9)	4.7 (2.1–9.1)	2.8 (1.4–5)
Centromere B	0 (0–4.8)	0 (0–5.4)	2.6 (0.3–9)	0.6 (0–3.2)	0.5 (0.1–1.8)
Sm	1.3 (0–7.2)	1.5 (0–8)	0 (0–4.6)	0 (0–2.2)	0.8 (0.2–2.2)
sm/RNP	3.7 (0.8–10.4)	10.5 (4.3–20.4)	7.4 (2.8–15.4)	8.7 (4.9–13.9)	7.6 (5.3–10.7)
Scl-70	0 (0–4.8)	0 (0–5.4)	0 (0–4.6)	1.8 (0.4–5.1)	0.8 (0.2–2.2)
Jo-1	0 (0–4.8)	0 (0–5.4)	1.3 (0–7.2)	0 (0–2.2)	0.8 (0.2–2.2)
DFS-70	1.3 (0–6.9)	0 (0–5.1)	0 (0–4.5)	0 (0–2.2)	0.8 (0.2–2.2)
**Antiphospholipid associated autoantibodies**					
**Cardiolipin**					
IgM	0 (0–4.6)	0 (0–5.3)	0 (0–4.5)	0.6 (0–3.4)	0.3 (0–1.4)
IgA	0 (0–4.6)	0 (0–5.3)	0 (0–4.5)	0 (0–2.2)	0 (0–1)
IgG	0 (0–4.6)	0 (0–5.3)	0 (0–4.5)	0.6 (0–3.4)	0.3 (0–1.4)
**β2-Glycoprotein1**					
IgM	0 (0–4.6)	0 (0–5.3)	0 (0–4.5)	0.6 (0–3.4)	0.3 (0–1.4)
IgA	0 (0–4.6)	0 (0–5.3)	0 (0–4.5)	0 (0–2.2)	0 (0–1)
IgG	0 (0–4.6)	0 (0–5.3)	0 (0–4.5)	0.6 (0–3.4)	0.3 (0–1.4)

CI, Confidence interval; ANA, Anti-nuclear antibodies; dsDNA, Double stranded-deoxyribonucleic acid; Ribosomal-P, Ribosomal protein; SSA/B, Anti-Sjögren’s-syndrome-related antigen A/B; Sm, Smith; RNP, ribonucleoproteins; Scl, Scleroderma 70; Jo, John.p; DFS, Dense fine speckled IgM/A/G, Immunoglobulin M/A/G.

Additional seroprevalences of both anti-infectious agents and autoantibodies, divided by regions, are detailed accordingly in Tables 2 and 3.

**Figure 2:**
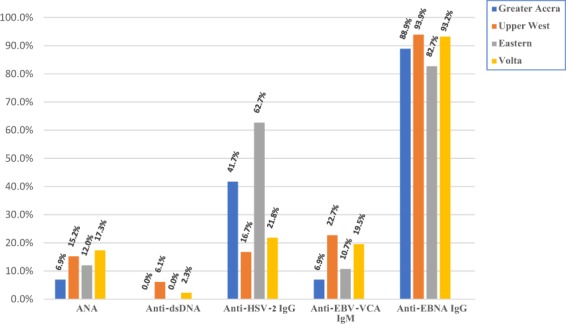
Distribution of seroprevalences of anti-infectious and autoantibodies between the different regions of Ghana. Abbreviations: ANA - Anti-nuclear antibodies, dsDNA - Double stranded deoxyribonucleic acid, EBV-VCA - Epstein-Bar virus viral capsid antigen, EBNA - Epstein-Bar virus nuclear antigen, HSV-2 - Herpes simplex Virus type 2, IgG/M, Immunoglobulin G/M.

Statistically significant differences in the seroprevalence of anti-EBNA IgG (p = 0.021), anti-EBV-VCA IgM (p = 0.008), anti-HSV-2 (p < 0.001) and anti-dsDNA (p = 0.048) antibodies was found between different regions (Fig. 2). The Eastern region had the lowest prevalence of anti-EBNA IgG antibodies and the highest prevalence of anti-HSV-2 antibodies, while the Upper West population had the lowest prevalence of anti-HSV-2 antibodies and the highest prevalence of anti-dsDNA antibody.

Investigation of the correlation between the seroprevalence of different antibodies showed lower ANA positivity among positive anti-HSV-2 individuals (p = 0.018; OR = 0.408; CI [0.19–0.85]). In the same manner, a lower seroprevalence of anti-dsDNA antibodies was found amid individuals positive for anti-HSV-2, but this finding was not statistically significant (p = 0.1).

With the purpose of clearing the above associations from confounding we investigated whether gender, age, and familial history of autoimmune diseases correlate to the seroprevalence of anti-HSV-2, anti-dsDNA or ANA. This analysis revealed that anti-HSV-2 antibodies were more prevalent in females (p = 0.001; OR = 2.254; CI [1.524–4.18]). Similarly, we found a statistically significant difference in ANA seroprevalence among the different age groups (p = 0.006), and a correlation between anti-HSV-2 antibodies and older age (p <= 0.001) was also noted.

Finally, while controlling for gender, age, region, and familial history of autoimmune diseases, the presence of anti-HSV-2 was found to be weakly inversely correlated with ANA positivity in the studied population (p = 0.044; adjusted OR = 0.398; CI [0.162–0.975]). Further results of the multivariable logistic regression analysis are summarized in Table 4.

**Table 4:** Multivariable logistic regression analysis including covariates associated with ANA prevalence.

Variable	Adjusted OR (95% CI)	P-value
**HSV2 IgG**		
Negative	1	
Positive	0.398 (0.162–0.975)	0.044*
**Age group (years)**		
18–30	1.255 (0.443–3.556)	0.669
31–40	0.280 (0.066–1.196)	0.086
41–50	0.335 (0.078–1.438)	0.141
51–60	0.657 (0.198–2.179)	0.492
≥*60*	1	
**Gender**		
Female	1	
Male	1.142 (0.560–2.328)	0.716
**Region**		
Greater Accra	1	
Upper West	1.399 (0.366–5.352)	0.624
Eastern	2.427 (0.666–8.837)	0.179
Volta	1.980 (0.613–6.399)	0.254
**AD-FHx**		
No	1	
Yes	1.023 (0.377–2.778)	0.964

OR, Odds-ratio; CI, Confidence interval; HSV2, Herpes simplex virus 2; IgG, immunoglobulin type G; Region 1, Greater Accra region; Region 2, Upper West region; Region 3, Eastern region; Region 4, Volta region; AD, Autoimmune disease; FHx, Family medical history.

* p < 0.05.

## Discussion

As research progresses, more and more pieces fall into place in the mosaic of autoimmunity, gradually forming a clearer picture towards understanding it. Yet, the transition from homeostasis into pathologic autoimmunity is still intangible, and it is likely that autoimmunity occurs as a continuum composed of various elements and risk factors, with autoimmune diseases at the very end of this spectrum^16^.

Highlighting the ambiguous nature of autoimmunity is the presence of naturally occurring antibodies in healthy subjects^17^. Autoantibodies may participate in the pathogenesis of autoimmune diseases such as myasthenia gravis or SLE, but their mere presence in the serum does not always suffice to induce autoimmune diseases^17^. Nevertheless, various studies suggest that the presence of autoantibodies might precipitate the development of autoimmunity^18,19^.

In the present study, we evaluated the prevalence of anti-infectious and autoantibodies in the Ghanaian population, as well as the possible association between these two types of antibodies.

We estimated the seroprevalence of ANA in Ghanaian healthy volunteers to be 14.3% (CI, 10.9–18.1%) and the seroprevalence of anti-dsDNA antibodies to be 2.3% (CI 1.1–4.3%).

Despite previous reports on the low prevalence of SLE in African countries^20^, the prevalence of both ANA and anti-dsDNA antibodies was compatible and even higher compared to previous published data from USA, Netherlands, Israel, Italy and Thailand^11,21^.

Furthermore, a previous study conducted on Ghanaians and Nigerians that emigrated to Italy found a very high prevalence of ANA (27.4%) which was even higher (35%) in those who had more recently arrived in Italy^22^. These intriguing data seem to indicate that ANA are indeed frequent in healthy West Africans and may disappear after many years of living in a Western country. In another small study done in Nigeria, the prevalence of ANA in healthy or hypertensive black African individuals was also very high (39.7%)^23^. The differences in ANA prevalence between different studies may be also attributed to different measuring methods of ANA positivity, such as immunofluorescence assay on HEp-2 cells, ELISA, or multiplex immunoassays.

Next, we sought out to differentiate the environmental and genetic pieces of the mosaic of autoimmunity. The fundamental role that environmental factors hold in autoimmunity is a well-established notion, even though the specific factors and the mechanisms involved in autoimmunity are still being established^24^. Supporting this notion are various epidemiological studies that found a gradient of autoimmune diseases prevalence which correlates with geographical latitude. The term “north-south gradient” represents this correlation and describes the rising incidence rates of autoimmune diseases further south and north of the equator^25^. Infections are considered a prominent environmental factor and some infectious agents have made a notorious reputation in the world of autoimmunity; EBV, HCV, *Streptococcus pyogenes* and many more have been associated with autoimmunity^26–28^.

In the present study, we observed a high prevalence of anti-EBV-VCA IgG antibodies, with total prevalence in all four districts reaching 99.2%. These findings resemble the prevalence of past EBV infection found in HIV carriers (87.2%) in a previous study by Adjei *et al*.^29^ but differ from the prevalence calculated among blood donors in the same study (20.0%)^29^. This disparity may be explained by the rigorous exclusion criteria from blood donors in Ghana, excluding intravenous drug users, chronically ill patients and donors classified with promiscuous sexual behavior or past infection with sexually transmitted diseases^29^. In addition, our findings of extremely high prevalence of EBV past infection are consistent with the global seroprevalence range of 80–90% in most countries^11,30,31^.

A very high prevalence of anti-HSV-1 antibodies was observed in our study population (96.7%, CI, 94.3–98.3%). These findings are consistent with previous epidemiological studies, estimating the prevalence of HSV-1 infection in sub-Saharan Africa at 87% between 0 and 49 years, with increasing prevalence through age^32^.

With regard to the correlation between autoantibodies and anti-infectious antibodies, we found an inverse correlation between ANA positivity and anti-HSV-2 positivity (OR = 0.408, CI [0.19–0.85]). This correlation remained marginally significant after adjusting for various factors such as gender, age, region, and familial history of autoimmune diseases; [OR = 0.403; CI [0.164–0.989]). Based on the demographical data of the study population, our initial assumption that region and ethnicity are highly associated led us to exclude ethnicity from the multivariable analysis. Another interesting observation was a trend towards lower prevalence of anti-dsDNA antibodies among anti-HSV-2 positive individuals (p = 0.1); however, likely due to the low prevalence of anti-dsDNA antibodies positive individuals, statistical significance was not reached.

To the best of our knowledge, previous studies have not revealed a clear connection between HSV-2 exposure and prevalence of ANA or anti-dsDNA antibodies. Additionally, the data regarding the prevalence of anti-HSV-2 antibodies in patients with autoimmune diseases such as SLE and multiple sclerosis is conflicting, with some studies finding high prevalence among autoimmune patients^33,34^, and others failing to find any statistical difference^35,36^. The weak inverse correlation between anti-HSV-2 antibodies and ANA and anti-dsDNA antibodies that was found in our study may hint that HSV-2 infection prior to the development of autoimmunity constitutes a protective factor.

The notion that infections might hold a protective effect against immune dysregulation was first introduced three decades ago, receiving the title ‘the hygiene theory’^37,38^. Supported by both epidemiological studies and animal models, evidence indicates a correlation between lower infectious burden and higher rate of autoimmune diseases such as type 1 diabetes mellites (T1DM) and multiple sclerosis. *Vice versa* is also correct, with exposure to a larger infectious load considered to be protective of autoimmunity, such as in the case of enrolment to day care in the first six months of life or presence of an older sibling^25,39^. The realization that infections might be beneficial against autoimmunity has led researchers to the identification of specific pathogens who possibly possess immunomodulatory attributes such as helminths, with helminth based pharmacological solutions being on their way^40,41^. Focusing on HSV-2, there are several possible mechanisms by which HSV-2 can extrapolate an immunomodulatory effect, such as induction of T-regulatory cells^42^. In a study by Milman *et al*. subjects previously infected with HSV-2 exhibited a significantly higher T-reg density in infected skin biopsies taken at reactivation and up to 8 weeks after lesion disappearance compared to unaffected skin^43^. Additionally, HSV-2 has also been shown to modulate dendritic cell activity, with evidence attesting to HSV-2 ability to prevent autophagosome formation, decrease T-cell activation via dendritic cells and reduce dendritic cells migration to lymph nodes^44^. Another immunomodulatory attribute associated with HSV-2 is the suppression of type 1-IFN signaling *via* inhibition of IFN promoter^45^. Lastly, and of particular relevance, HSV-2 has been shown to suppress antibody formation in response to specific pathogens in a murine model^46^, indicating that HSV-2 could potentially inhibit expression of specific autoantibodies.

One limitation of the present study is a possible selection bias, with serum samples obtained from volunteers responding to an advertisement. For example, 32% of our study population belonged to the 18–30 years old group. In addition, this study did not include all the regions of Ghana, and further studies are required in this regard. Furthermore, future studies may also consider evaluating antibodies against other viral and bacterial pathogens.

In conclusion, the present study revealed a high prevalence of anti-EBV and anti-HSV-1 antibodies in Ghanaian population that reached an estimated prevalence of 99.2% and 96.7% respectively. Remarkably, a new possible association was revealed between HSV-2 infection and low seroprevalence of ANA, suggesting a protective trait of HSV-2 infection against autoimmunity, which is also supported by a trend towards decreased prevalence of anti-dsDNA antibodies among subjects exposed to HSV-2. This previously undescribed association needs further characterization.
